# Relative specificity as an important consideration in the big data era

**DOI:** 10.3389/fgene.2022.1030415

**Published:** 2022-10-06

**Authors:** Xiaoxiao Zhang, Yan Zeng

**Affiliations:** Department of Zoology, College of Life Sciences, Nanjing Agricultural University, Nanjing, China

**Keywords:** relative specificity, big data, genomics, complex biochemical system, microRNA

## Abstract

Technological breakthroughs such as high-throughput methods, genomics, single-cell studies, and machine learning have fundamentally transformed research and ushered in the big data era of biology. Nevertheless, current data collections, analyses, and modeling frequently overlook relative specificity, a crucial property of molecular interactions in biochemical systems. Relative specificity describes how, for example, an enzyme reacts with its many substrates at different rates, and how this discriminatory action alone is sufficient to modulate the substrates and downstream events. As a corollary, it is not only important to comprehensively identify an enzyme’s substrates, but also critical to quantitatively determine how the enzyme interacts with the substrates and to evaluate how it shapes subsequent biological outcomes. Genomics and high-throughput techniques have greatly facilitated the studies of relative specificity in the 21st century, and its functional significance has been demonstrated in complex biochemical systems including transcription, translation, protein kinases, RNA-binding proteins, and animal microRNAs (miRNAs), although it remains ignored in most work. Here we analyze recent findings in big data and relative specificity studies and explain how the incorporation of relative specificity concept might enhance our mechanistic understanding of gene functions, biological phenomena, and human diseases.

## Introduction

For a gene to fulfill its functions, the gene product, i.e., a protein, RNA, or enzyme, must interact with proteins, RNAs, DNA elements, or other molecules, so characteristics of those interactions underline the mechanism. Use the enzyme:substrate relationship for analogy, the enzyme and substrate entities referring to interacting partners with the former being the party inducing conformational, catalytic, and/or functional changes in the latter. It is clear then that an enzyme seldom has only one substrate, and it is also intuitive that if an enzyme has multiple substrates, it will not interact with them equally. This phenomenon has been defined as relative specificity and generalized by the relative specificity hypothesis, which proposes that quantitatively different enzyme:substrate reactions have functional significance through impacting the substrates selectively and, hence, regulating the underlying biological processes and phenotypes (Zeng, 2011).

Relative specificity is apparent in simple systems. For example, human hemoglobin binds O_2_, CO_2_, and CO, and CO associates much tighter than O_2_ with hemoglobin, which has a profound physiological implication. On the other hand, biology is dominated with complex enzymes and systems, such as transcriptional factors, RNA-binding proteins, protein kinases, which have hundreds or more substrates and interacting partners, and the ribosome and RNA polymerases with thousands or tens of thousands of substrates. Studying their relative specificity has been traditionally hampered by technological limitations and the ensuing avoidance of the subject (Zeng, 2011). Prior to genome sequencing, it was impossible to know the target range of a transcription factor. With genome sequences emerging since the 1990s, painstaking work must be carried out to screen for and confirm the hundreds or thousands of substrates for any enzyme, while comparing their interactions with the enzyme requires development of the suitable assays, e.g., *in vitro* translation, which might not be available or easy to scale up. Lastly, the substrates or products *in vivo* must be quantified at a large scale using methods such as high-throughput proteomics whose sensitivity and accuracy is still being improved. Despite lingering difficulties, genomics techniques in the past 10–20 years have nonetheless opened up new venues of research to reveal instances of relative specificity having functional consequences in diverse settings.

## Evidence in support of the relative specificity hypothesis

Due to its early application of high-throughput approaches, the transcription field has accumulated the most data from which relative specificity can be deduced. *S. cerevisiae* transcription factor Ndt80 varies in affinities for target promoter sequences, which explains differential gene activation at the exact times during sporulation (Zeng, 2011). Studies in animals including *Drosophila* indicate that transcription factors bind and control their target genes with a mechanism best described as “quantitative continua” (Biggin, 2011). Chromatin immunoprecipitation followed by sequencing (ChIP–seq) experiments have shown that transcription factors bind thousands of target genes variably, and transcription activator binding positively associates, while transcription repressor binding negatively associates, with human target mRNA levels (Zeng, 2014). Thus, merely by differential binding to targets a transcription factor can modulate the expression of thousands of genes directly.

**FIGURE 1:**
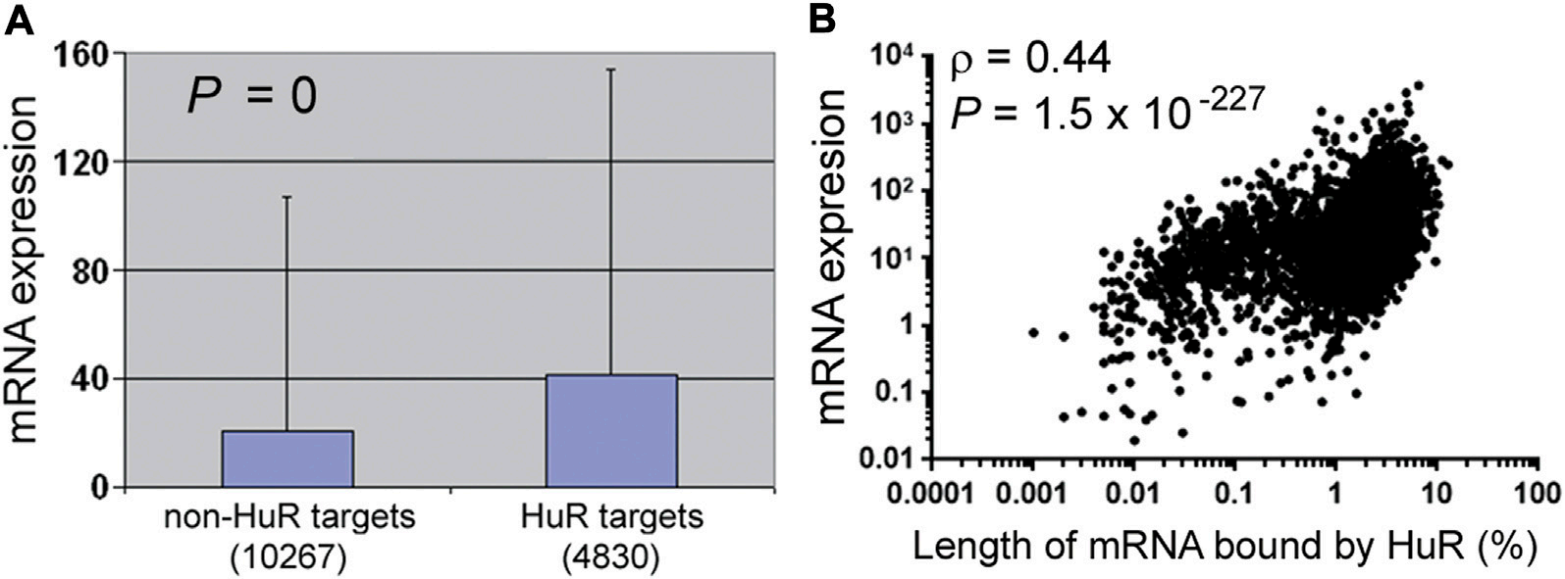
Effects of HuR binding on RNA expression in 293T cells. **(A)** Comparisons of the RNA expression levels of non-HuR targets and HuR targets [GSE29943 and Supplementary Table S2 from (Lebedeva et al., 2011)]. RNAs with fragments per kilobase of transcript per million mapped reads above 0 (the *y*-axis) were counted, and the numbers of RNAs in each category are listed in parentheses. Mann–Whitney *U* test (SPSS, IBM, Armonk, New York) was performed to compare RNA expression, and the graph depicts the averages, standard deviations, and the *p* value. Corresponding to the data of Supplementary Table S1. **(B)** Spearman correlation analysis between HuR binding [the *x*-axis, Supplementary Table S2 from (Lebedeva et al., 2011)] and RNA expression (the *y*-axis, GSE29943). Dots represent individual HuR targets, and the correlation coefficient (ρ) and *p* value shown in the graph. Corresponding to the data of Supplementary Table S2.

RNA-binding proteins regulate RNA metabolism and functions. YTHDF proteins bind m^6^A in mRNAs, and how much mRNAs are degraded varies with the numbers of m^6^A sites (Zaccara and Jaffrey, 2020). Likewise, the amount of RBFOX1 binding positively correlates with the abundance of its target mRNAs (Fogel et al., 2012; Ray et al., 2013). Another example is HuR, whose RNA targets in human 293T cells were analyzed by HuR antibody pull-down and sequencing (Lebedeva et al., 2011; Mukherjee et al., 2011; Zeng, 2011). HuR targets are overall more abundant than the non-targets, supporting the well-established role of HuR in RNA stabilization (Figure 1A). Critically, HuR targets also vary in the amount of HuR binding, and the more HuR binds, the higher the RNA is expressed (Figure 1B). That a single protein accounts for 19% (0.44^2^) of the differential expression of over 4,000 RNAs underscores the importance of relative specificity.

Protein kinases are another class of enzymes for which relative specificity has been demonstrated. The mammalian mTORC1 kinase phosphorylates some peptides/substrates better than others, a sign of relative specificity or substrate quality, allowing mTORC1 to control growth and rapamycin sensitivity (Kang et al., 2013). The most thoroughly dissected example is Cdk1 of the fission yeast *S. pombe* (Swaffer et al., 2016; Basu et al., 2022). Cdk1 also has good and poor substrates. Its activity steadily increases from the G1 to M phase during the cell cycle, when it is able to phosphorylate only the good substrates early on, which promote DNA replication, and the poor ones later, which promote cytokinesis. If Cdk1 phosphorylates the poor substrates prematurely, yeast will divide without doubling its DNA and subsequently die, a serious outcome should relative specificity fail. Thus, fluctuating Cdk1 activities largely drive the cell cycle progression because relative specificity mandates Cdk1 to phosphorylate functionally distinct substrates at separate, appropriate times during the cell cycle (Basu et al., 2022).

The textbook version of the ribosome consists of a fixed stoichiometry of the same ribosomal RNA and protein components and operates like an assembly-line machine. This simplistic view has been overturned (Xue and Barna, 2012; Briggs and Dinman, 2017). RNA and protein composition of the ribosome can change, with physiological and disease relevance (Kiparisov et al., 2005; Kondrashov et al., 2011; Farley and Baserga, 2016; Shi et al., 2017). And the ribosome translates different mRNAs at different rates. Certain N-terminal codons in bacterial mRNAs form a weak secondary structure to favor ribosome binding and enhance translation initiation (Bentele et al., 2013; Goodman et al., 2013). After initiation, translation elongation is governed by codon optimality, an index of mRNA codon recognition by tRNAs (Hanson and Coller, 2018; Bae and Coller, 2022). Codon optimality even impacts mRNA degradation, according to global mRNA sequence and expression correlation analyses (Hanson and Coller, 2018; Carneiro et al., 2019; Bae and Coller, 2022). The ribosome, therefore, by its nature of relative specificity regulates both protein and mRNA expression. It must be noted, however, that codon optimality remains an indirect marker of translation elongation, and how it controls global translation independent of its effect on mRNA stability has not been evaluated; previous studies examined only select mRNAs and their codon mutants (Presnyak et al., 2015).

Relative specificity has also been identified for Hsp90 (Taipale et al., 2012) and animal miRNAs (discussed later). Together these studies emphasize that to understand an enzyme’s work, we not only need to seek out its substrates, but also must recognize that it interacts with substrates with different propensities, which is fully integrated into this enzyme’s mechanism and function. Unfortunately, we have not achieved this level of understanding for most complex systems, as explicit evidence of relative specificity remains scarce, especially the direct demonstration of its physiological significance.

## Challenges remain in the big data era

Genomics and high-throughput techniques have removed the technical obstacles in studying complex systems, yet the number of instances where relative specificity has been tested pales in comparison to the candidate pool: only a handful of kinases and RNA-binding proteins have been examined, whereas thousands exist in humans. ChIP-seq data show uneven deposition of DNA-binding proteins to genes, but rarely do they entice an examination of how genome-wide transcription is controlled as a result. This oversight might stem from the thinking that eukaryotic transcription requires the binding of an avalanche of transcription factors such that the contribution of any single protein is buried. Moreover, while genomics screening is now routine and typically yields hundreds of hits, most researchers would then pick a handful of hits for subsequent investigation based on expression changes or gene functions. This drastic target narrowing is adopted because experimentation with individual genes is time and effort intensive, yet there lies another reason: the under-appreciation of relative specificity, even in this big data era. Consequently, the information about relative specificity is not collected, discounted, or discarded by target triage. The goal of big data is to dissect and model biological systems comprehensively, but without relative specificity, any conclusions or models will lack vital information and are substantially weakened.

Below we use three fundamental subjects to explain the importance of pursuing relative specificity. The first pertains to gene functions in general and in disease. While a gene, e.g., *BRCA1*, may be expressed in multiple tissues and organs and have critical, house-keeping functions, why do its germline mutations often affect only a specific organ(s), e.g., the breasts and ovaries? And how do mutations in genes, e.g., *TP53* and *MYC* which have a lot of targets, cause cancers? To the first question, an organ-specific factor may relay signals from germline mutations to trigger disease in that particular organ. Consistent with this view, a new study identified Pax8, a kidney-enriched transcription factor, as a downstream effector of *VHL* mutations in renal carcinogenesis (Patel et al., 2022). Tissue-specific factors are undoubtedly essential, yet they fit only part of the picture. Pax8 is highly expressed in the thyroids and kidneys, but it is also present in other organs, so more work is needed to understand how Pax8 works with the ubiquitous *VHL* in those organs. Furthermore, Pax8 is a transcription factor and counts *MYC* as a target (Patel et al., 2022). Thus, this paper pushes the organ-specificity goalpost down the field while circling back to the second question above: how exactly do proteins including transcription factors that control so many downstream genes cause cancers? A great deal of transcription target genes of *TP53* and *MYC* have been identified and studied for decades, but not a single one mediates or recapitulates the effects of *TP53* or *MYC* mutations. From the relative specificity viewpoint, mutations in *TP53*, *MYC,* or other genes might induce unbalanced changes in the targets, and it might be the targets’ unbalanced changes that cause the diseases. So a new research angle is to quantify the relative levels or changes in many targets: the identity of individual genes still matters, just more must be considered together.

The second subject is transcription. A recent study cloned tens of millions of random 80-basepair-long DNA sequences into the promoter region of a yellow fluorescence protein reporter gene and measured reporter expression in *S. cerevisiae*, from which sequence-to-expression models were built (Vaishnav et al., 2022). The authors then asked how gene regulatory DNA evolved by coupling library sequences to the natural promoters of 5,569 *S. cerevisiae* genes in 1,011 *S. cerevisiae* isolates and comparing gene expression changes (Vaishnav et al., 2022). This paper illustrates perfectly how big data have empowered massive sequence space expansion and gigantic parallel reporter assays to greatly facilitate measuring relative specificity, and it yields an impressive amount of data for reporter gene regulation. It does not, however, answer why, from a genome perspective, endogenous genes A and B are expressed differentially in yeast. A wide assortment of genomics, epigenetics, transcriptomics, and ChIP-seq techniques have been developed to study gene regulation from yeast to humans. As genes vary in their amounts of transcription factor binding and DNA and chromatin modifications, the challenge is to combine and model DNA sequences, modifications, chromatin structures, and transcription factor binding systematically, injecting relative specificity at each step.

**FIGURE 2:**
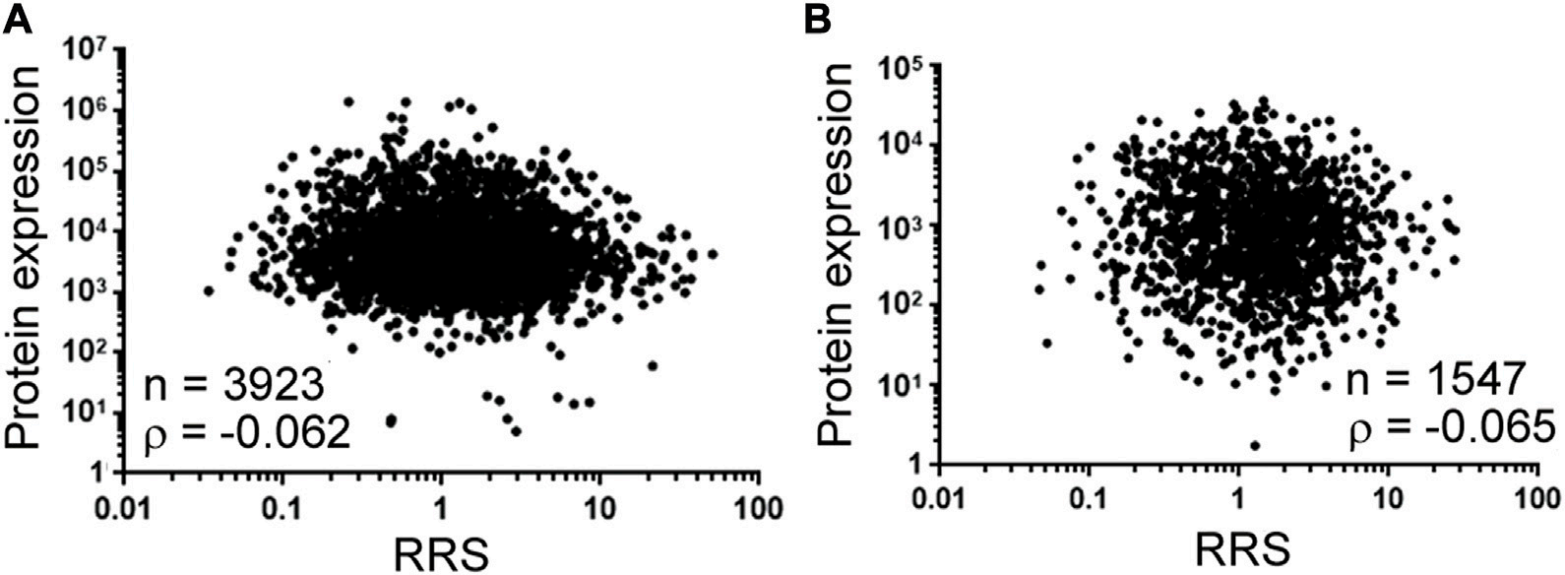
Ribosome recruitment (RRS) and protein expression in yeast. **(A)** Spearman correlation analysis between RRS [the *x*-axis, according to dataset GSE182290 (Niederer et al., 2022)] and protein expression (the *y*-axis) from Supplementary Table S4 in (Ho et al., 2018). Dots represent individual genes (mRNAs and the corresponding proteins). For mRNAs with isoforms, we added their RRS prior to correlation analysis. Number of genes (n) and the correlation coefficient (ρ) are shown on the graph. Corresponding to the data of Supplementary Table S3. **(B)** Spearman correlation between RRS (the *x*-axis) (Niederer et al., 2022) and protein expression (the *y*-axis) from Supplementary Table S4 in (Lahtvee et al., 2017). Labeling and descriptions see **(A)**. Corresponding to the data of Supplementary Table S4.

The last subject is translation. Differential translation has been investigated using bioinformatics and reporter libraries, but direct, large-scale validation in endogenous protein expression has rarely been performed. A latest study constructed a library of thousands of natural mRNA sequences from 4,252 *S. cerevisiae* genes, and incubated the mRNAs consisting of up to 122-nucleotide-long 5’ untranslated regions in front of at least 24-nucleotide-long coding sequences with yeast extract for *in vitro* translation initiation reactions (Niederer et al., 2022). The authors then sequenced ribosome-bound mRNAs and found that ribosome recruitment (RRS) to mRNAs differed by 1,000-fold. The obvious next question is: is recruitment reflected in protein levels *in vivo*? It was not documented in the paper, so we analyzed the correlation of RRS to protein expression in yeast (Figure 2). Figure 2A used the yeast protein expression data that summarized the results of 21 previous genome-wide studies (Ho et al., 2018), and Figure 2B used the protein expression data normalized to mRNA data, which reduced the influences from mRNA abundance (Lahtvee et al., 2017). Both indicated a correlation close to 0, suggesting that ribosome recruitment leaves no mark on global protein expression in yeast. This result underlines two principal missing pieces in relative specificity investigation: one is the lack of demonstration of physiological relevance in the literature, and the other is that an enzyme interacting with its substrates differently is not necessarily reflected in the end products. Thus, even if relative specificity is self-evident biochemically, its physiological importance is never certain.

Altogether, the above examples suggest that more evidence of relative specificity remains to be unearthed, and that we need a deeper understanding of its biological relevance. In pathways that involves multiple enzymes or steps, e.g., translation initiation, elongation, and termination, is there a step whose relative specificity is the most critical? As there is often a rate-limiting reaction in metabolism and cell signaling, is there a rate-limiting relative specificity? Lastly, if relative specificity is a general principle, is it evolutionarily conserved? Studies in animal miRNAs have begun to address these questions.

## Lessons from the mirna system

miRNAs are a family of approximately 22-nucleotide-long RNAs that are widely expressed in metazoans, and they regulate biological processes by binding to complementary sequences, usually in the 3’ untranslated regions, in target mRNAs to repress their expression (Bartel, 2018). miRNA biogenesis starts with transcription to produce primary miRNA transcripts (pri-miRNAs). During canonical animal miRNA processing, the ribonuclease Drosha/DGCR8 (Drosha in short) cleaves pri-miRNAs to generate precursor miRNAs (pre-miRNAs) in the nucleus. Next Exportin5 exports the pre-miRNAs to the cytoplasm, where another ribonuclease Dicer cleaves pre-miRNAs to produce miRNA duplex intermediates. Argonaute proteins then select the mature miRNA strands, and the miRNA:Argonaute complexes bind target mRNAs to repress gene expression. As animal genomes encode hundreds of miRNAs, and each miRNA has hundreds of targets (Bartel, 2018; Kozomara et al., 2019), both miRNA biogenesis and miRNA function constitute complex systems in which relative specificity can be tested.

We found that human Drosha cleaves hundreds of pri-miRNA substrates at different rates *in vitro*, which correlates with mature miRNA expression *in vivo*, revealing a role of Drosha’s relative specificity in regulating miRNA biogenesis (Feng et al., 2011). Human Dicer also cleaves pre-miRNAs differentially *in vitro*, as does human Exportin5 bind pre-miRNAs, yet there is no significant impact on miRNA expression associated with Dicer or Exportin5 action (Feng et al., 2012; Zhang et al., 2021). A rationale may be that because pri-miRNA cleavage by Drosha signifies the first and irreversible step in processing, it makes sense that Drosha selectivity dominates; i.e., there may indeed be a rate-limiting relative specificity (Zeng, 2014). Secondary structural features in pri-miRNAs and pre-miRNAs important for differential interactions with Drosha, Dicer, and Exportin5 were identified, providing mechanistic explanations to how relative specificity arises (Feng et al., 2011; Feng et al., 2012; Zhang et al., 2021). Differential transcription of miRNA genes has a lower correlation coefficient with miRNA expression than Drosha (Feng et al., 2011; Zhang et al., 2018), suggesting that processing might play a more prominent, regulatory role genome-wise.

miRNA function similarly exhibits relative specificity. In cell culture models miRNAs such as miR-124 inhibit reporter genes containing the 3’ untranslated regions of approximately 200 target mRNAs to different degrees, and such differential inhibition correlates with the expression levels of the corresponding, endogenous target mRNAs in relevant human tissues (Li et al., 2019). This result suggests that miRNA action contributes to differential target expression. Targeting efficacy by miRNAs has also been examined using artificial libraries without further analyses of the functional relevance *in vivo* (Vainberg Slutskin et al., 2018; Becker et al., 2019; McGeary et al., 2019).

Lastly, is relative specificity conserved through evolution? To answer this question cleavage of zebrafish and fruitfly pre-miRNAs and pri-miRNAs by Dicer and Drosha, respectively, were examined (Zhang et al., 2022). Both Dicer and Drosha discriminate their substrates *in vitro*, but in both animals only the preference of Drosha correlates with global, differential miRNA expression *in vivo*, just as in humans. Hence, relative specificity of the enzymes is evolutionarily conserved, so is, crucially, their relative contribution to the regulation of global miRNA production.

Studies of miRNAs and other, completely different systems have converged on similar findings. Both Drosha and fission yeast Cdk1 have essential targets and perform essential functions, and neither acts like production-line robots. By intrinsic biochemical properties, Drosha ensures that miRNAs are generated at varying amounts, thereby regulating miRNAs, miRNA targets, and processes downstream (Feng et al., 2011; Zhang et al., 2022), and Cdk1 ensures that proper substrates are phosphorylated at proper times, maintaining an orderly cell cycle (Swaffer et al., 2016; Basu et al., 2022). Most publications on miRNA targets are content with analyzing a single target and its contribution to a phenotype, yet a miRNA has many targets (Bartel, 2018; Kozomara et al., 2019; Li et al., 2019). Focusing on a single target will not provide an adequate answer to miRNA functions because as a miRNA has many targets, it is quite probable that some have opposing roles in a particular pathway. Without knowing how the miRNA represses those targets comparatively, how can one ascertain the “net” outcome and the mechanism? Obviously the same reasoning applies broadly to transcription factors and other proteins and enzymes with many substrates.

## Concluding remarks

Relative specificity and its physiological importance have been documented in a tiny number but wide range of complex biochemical systems that have been sampled, and we have gained novel and unexpected insights into how biological processes are regulated. What is the origin of relative specificity? As it describes the interactions between molecules, the same physics and chemistry principles apply. In the earliest days when there were only one enzyme and one substrate, no relative specificity existed. But as the biological world increased in diversity, more substrates with different sequences and structures emerged, which naturally diverged in their interactions with the enzyme. Relative specificity might highlight a new layer of controlling mechanisms and complexity, as natural selection could act on relative specificity in addition to the more visible, sequence space to maintain stability while fostering evolution at the same time.

As important as relative specificity is, it works alongside with other regulatory mechanisms. *In vitro* studies cannot capture the intricacy that the levels of enzymes and substrates may fluctuate temporally and spatially, between and within cells (Raj and van Oudenaarden, 2008). For example, miRNA function depends on miRNA:target affinities as well as the abundance of miRNAs and individual mRNAs (Bosson et al., 2014; Denzler et al., 2014; Nam et al., 2014; Li et al., 2019). Many biological systems are under weak constraints, with components evolving under largely neutral selection. Consequently, networks can be stochastic yet robust and tolerate variations due to relative specificity and/or other mechanisms (Lynch, 2007; Raj and van Oudenaarden, 2008; Paris et al., 2013). These complexities might obscure the effects of relative specificity (Csardi et al., 2015), but together they call for a more quantitative research approach.

In summary, new technologies and studies in the 21st century have generated massive data, now further aided by artificial intelligence to study complex systems and diseases. But these efforts have pitfalls because in most cases the information about relative specificity is missing or insufficiently utilized. Fortunately, relative specificity can be studied with current technologies and the right mindset, and its incorporation in the big data era will help us better understand and model complex systems.

## Data Availability

The original contributions presented in the study are included in the article/Supplementary Material, further inquiries can be directed to the corresponding author.
